# Prediction of treatment response after stereotactic radiosurgery of brain metastasis using deep learning and radiomics on longitudinal MRI data

**DOI:** 10.1038/s41598-024-60781-5

**Published:** 2024-05-15

**Authors:** Se Jin Cho, Wonwoo Cho, Dongmin Choi, Gyuhyeon Sim, So Yeong Jeong, Sung Hyun Baik, Yun Jung Bae, Byung Se Choi, Jae Hyoung Kim, Sooyoung Yoo, Jung Ho Han, Chae-Yong Kim, Jaegul Choo, Leonard Sunwoo

**Affiliations:** 1 Department of Radiology, Seoul National University Bundang Hospital, Seoul National University College of Medicine, 82, Gumi-Ro 173Beon-Gil, Bundang-Gu, Seongnam, Gyeonggi 13620 Republic of Korea; 2 Kim Jaechul Graduate School of Artificial Intelligence, KAIST, 291 Daehak-Ro, Yuseong-Gu, Daejeon, 34141 Republic of Korea; 3 Letsur Inc, 180 Yeoksam-Ro, Gangnam-Gu, Seoul, 06248 Republic of Korea; 4 Office of eHealth Research and Business, Seoul National University Bundang Hospital, 82, Gumi-Ro 173Beon-Gil, Bundang-Gu, Seongnam, Gyeonggi 13620 Republic of Korea; 5 Department of Neurosurgery, Seoul National University Bundang Hospital, Seoul National University College of Medicine, 82, Gumi-Ro 173Beon-Gil, Bundang-Gu, Seongnam, Gyeonggi 13620 Republic of Korea; 6 Center for Artificial Intelligence in Healthcare, Seoul National University Bundang Hospital, 82, Gumi-Ro 173Beon-Gil, Bundang-Gu, Seongnam, Gyeonggi 13620 Republic of Korea

**Keywords:** Brain metastasis, Stereotactic radiosurgery, Deep learning, Radiomics, Longitudinal analysis, Treatment response, Cancer, Neuroscience, Oncology

## Abstract

We developed artificial intelligence models to predict the brain metastasis (BM) treatment response after stereotactic radiosurgery (SRS) using longitudinal magnetic resonance imaging (MRI) data and evaluated prediction accuracy changes according to the number of sequential MRI scans. We included four sequential MRI scans for 194 patients with BM and 369 target lesions for the Developmental dataset. The data were randomly split (8:2 ratio) for training and testing. For external validation, 172 MRI scans from 43 patients with BM and 62 target lesions were additionally enrolled. The maximum axial diameter (Dmax), radiomics, and deep learning (DL) models were generated for comparison. We evaluated the simple convolutional neural network (CNN) model and a gated recurrent unit (Conv-GRU)-based CNN model in the DL arm. The Conv-GRU model performed superior to the simple CNN models. For both datasets, the area under the curve (AUC) was significantly higher for the two-dimensional (2D) Conv-GRU model than for the 3D Conv-GRU, Dmax, and radiomics models. The accuracy of the 2D Conv-GRU model increased with the number of follow-up studies. In conclusion, using longitudinal MRI data, the 2D Conv-GRU model outperformed all other models in predicting the treatment response after SRS of BM.

## Introduction

The incidence of brain metastasis (BM) ranges from 10 to 40% in adult patients with cancer^1,2^. Traditionally, whole-brain radiation therapy (WBRT) has been the primary treatment for patients with multiple BMs. However, because of the risk of cognitive decline associated with WBRT and the improved detection rate of small BMs using three-dimensional (3D) magnetic resonance imaging (MRI), stereotactic radiosurgery (SRS) has become more prevalent in patients with oligometastases^3–5^.

The treatment response of BM is typically evaluated based on changes in the sum of the longest diameter of the enhancing lesions using the Response Assessment in Neuro-Oncology Brain Metastasis (RANO-BM) criteria^6^. However, after SRS, clinicians often encounter an increase in the tumour size or the appearance of new contrast-enhancing lesions. In such cases, it may be impossible to differentiate between post-treatment changes and tumour progression using the RANO-BM criteria because of this transient increase in size. In addition, radiation necrosis may develop, and the size of the lesion may continue to increase, further complicating the problem. This phenomenon puts clinicians in a great dilemma, as it can be difficult to distinguish precisely between two radiologically similar but distinct conditions—radiation necrosis and tumour progression—in the early post-treatment period^7^. Therefore, confirming the treatment response may require a long-term follow-up period, which can delay early intervention^8^.

To address this issue, researchers have attempted to differentiate the two conditions by utilising advanced MRI techniques and/or artificial intelligence (AI); however, they have been unsuccessful thus far^8–17^. Moreover, most AI studies examining this issue have employed radiomics as a method^18^, with only a few studies applying deep learning (DL) based on MR images from a single time point, which only demonstrated modest performance^17^. No previous studies have utilised MR images from multiple time points to assess the treatment response of BM.

Hence, we aimed to develop AI models for predicting the treatment response after SRS using longitudinal data. We developed three different models (maximum axial diameter [Dmax], radiomics, and DL) based on MR images from four sequential time points (one pre-treatment and three post-treatment) and compared their performances. Additionally, we aimed to evaluate the change in prediction accuracy according to the number of sequential MRI scans to identify the optimal number of follow-up scans. Furthermore, we conducted an external validation using an independent dataset to assess the generalisability of our model.

## Methods

This retrospective study was reviewed and approved by our institutional review board (Seoul National University Bundang Hospital IRB No. B-2012-652-109), which waived the requirement for informed consent for data evaluation. We ensured that all images were anonymised prior to download. Furthermore, any extraneous patient information was blinded and managed using a unique research identifier to uphold patient privacy and data security. The results are reported in accordance with the relevant reporting guidelines or recommendations specified for AI research using medical data^19–21^. All MRI Digital Imaging and Communications in Medicine files were anonymised and de-identified before the analysis.

### Patient selection

We retrospectively reviewed the medical data of patients with BM between January 2015 and October 2020 for the Developmental dataset. The patients were selected based on the following inclusion criteria: (a) age > 19 years; (b) patients with proven underlying malignancy as a primary source; (c) patients diagnosed with BM with a high likelihood using brain MRI; (d) presence of a precise date of SRS for the BM; (e) underwent baseline MRI on the same date as SRS (pre-SRS); (f) underwent follow-up MRI at least three times after SRS with intervals of > 30 days (first to third post-SRS follow-up); (g) and were followed-up clinically and radiologically after the third follow-up MRI to assess the treatment response of SRS. The exclusion criteria were as follows: (a) history of brain surgery before SRS, (b) history of WBRT before SRS, (c) absent 3D post-contrast T1-weighted images with 1-mm slice thickness from pre-SRS or follow-up MRI, or (d) visible BM nodules < 5 mm on pre-SRS MR images.

For external validation, we additionally enrolled patients with BM between November 2020 and December 2022, adhering to the same inclusion and exclusion criteria established for the Developmental dataset. Given the temporal separation in MRI acquisition dates relative to the Developmental dataset, we designated this dataset as the Temporal test set.

### MRI examination

MRI examinations were performed using a 1.5-T (Intera, Philips Healthcare, Best, Netherlands; and Magnetom Amira, Siemens, Germany) or 3.0-T scanner (Achieva, Ingenia, or Elition, Philips Healthcare; and Vida, Siemens) with an 8- or 32-channel head coil. The MRI parameters for the 3D gradient echo sequence were as follows: field of view (FOV), 240 × 240 mm^2^; acquisition matrix, 240 × 240; slice thickness, 1 mm; number of excitations, 1; repetition time (TR), 8–10.6 ms; echo time (TE), 3.7–5.7 ms; and flip angle, 8°. The MRI parameters for the 3D turbo spin-echo sequence with the black blood technique were as follows: FOV, 240 × 240 mm^2^; acquisition matrix, 240 × 240; slice thickness, 1 mm; number of excitations, 1; TR, 500 ms; TE, 30 ms; and flip angle, 90°. For contrast enhancement, gadobutrol (Gadovist^®^, Bayer Schering Pharma AG, Berlin, Germany; 0.1 mmol/kg) was injected intravenously.

### MRI analysis

For the Developmental dataset, we included 194 patients with 369 target BM lesions from 776 MRI examinations (four MRI scans per patient, including one pre-SRS and three post-SRS MRI scans). The data were divided randomly into training and testing datasets in a ratio of 8:2. For the training set, we used 616 MRI scans from 154 patients. For the testing set, we used 160 MRI scans from 40 patients. For the Temporal test set, we included 43 patients with 62 target BM lesions from 172 MRI examinations. We defined measurable disease as a contrast-enhancing lesion that could be measured accurately in at least one dimension with a minimum size of 5 mm (modified RANO-BM criteria). The size threshold of the modified RANO-BM criteria is smaller than that of the RANO-BM criteria (10 mm). This modification was suggested by the RANO-BM working group only in the setting of brain MRI with a slice thickness ≤ 1.5 mm^6^. Otherwise, the modified RANO-BM criteria follow the RANO-BM criteria^6^. The maximum diameter of each BM was measured on the representative axial plane. Two neuroradiologists (S.J.C. and L.S. with 9 and 12 years of experience in neuroradiology, respectively) assessed the ground truth for treatment response according to the modified RANO-BM criteria by consensus. Upon determining the ground truth, the reviewers had access to all clinical information and follow-up MRI scans after the third post-SRS MRI. The histopathological results were used to establish ground truths for BM nodules verified through surgery. The treatment response was dichotomised into progressive disease (PD) versus non-PD; complete response, partial response, and stable disease were classified as non-PD^6^. The regions of interest in all BM nodules were semi-automatically drawn along the enhancing tumour margin by two neuroradiologists by consensus using AI-based commercial software (MediLabel®, Ingradient, Republic of Korea)^22^.

### Model development for comparison

**Figure 1:**
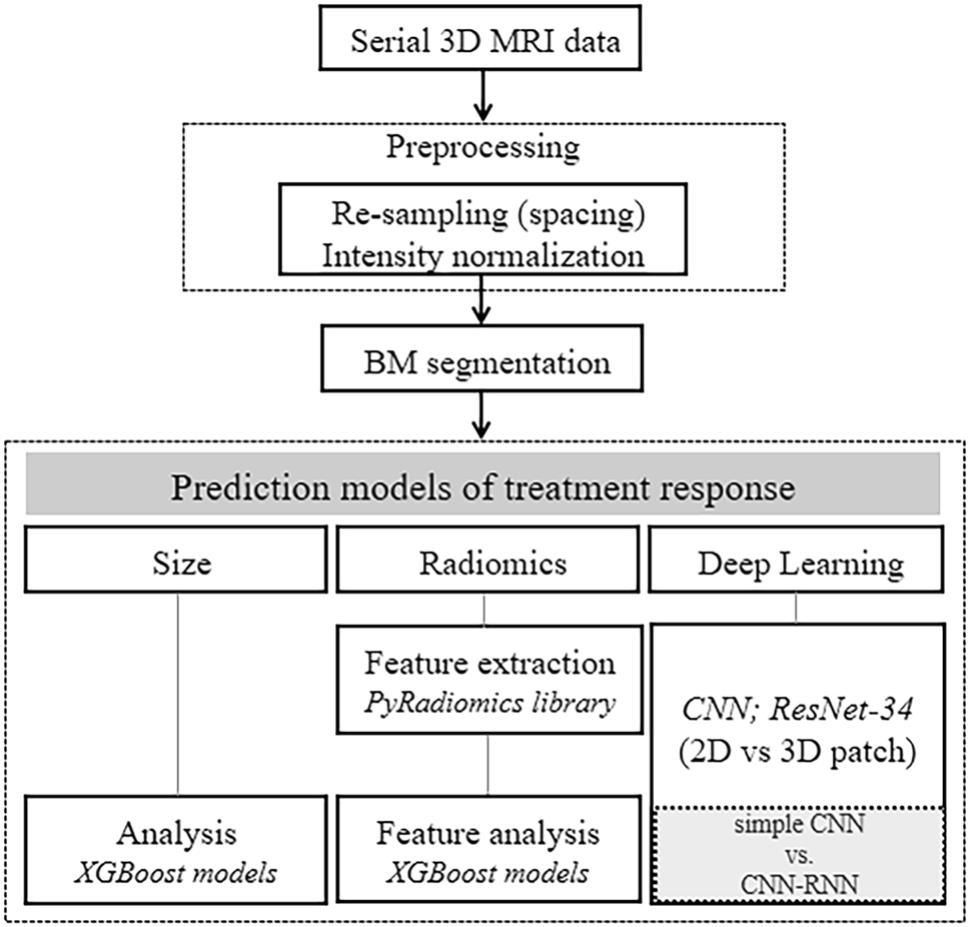
Flowchart of the proposed deep learning-based computer-aided detection system. *BM* brain metastasis, CNN convolutional neural network, *RNN* recurrent neural network, *2D* two-dimensional, *3D* three-dimensional.

We developed three arms to compare the performance of treatment response prediction: Dmax, radiomics, and DL. The common processes for arm development included pre-processing, BM segmentation, feature extraction, and sequential modelling. In sequential modelling, we employed machine learning algorithms, capable of capturing significant temporal patterns and feature importance without manual feature engineering. We performed end-to-end prediction modelling for the DL arm, sequential feature extraction and analysis modelling for the radiomics arm, and modelling for the Dmax arm (Fig. 1).

In the pre-processing step, each voxel's spacing and signal intensity on the MR image varied based on the scan parameters. Thus, we resampled the image to obtain a voxel spacing of 0.5 × 0.5 × 0.5 mm^3^. Subsequently, we normalised the image by resampling its signal, excluding the background, which ranged from -1 to 1 based on the signal intensity of the position manually selected in the grey matter. To extract the subregions containing a single BM, we cropped the image into 3D patches with a size of 64 × 96 × 96 voxels based on the BM segmentation labels provided by the mentioned neuroradiologists.

An NVIDIA GeForce GTX 1080 Ti graphics processing unit (NVIDIA, Santa Clara, CA, USA) was used for DL. Furthermore, DL training was conducted using Python 3.8.10 and the PyTorch 1.6.0 framework in the Ubuntu 16.04.6 operating system. We used the PyCharm (JetBrains s.r.o., Prague, Czech Republic) and Visual Studio Code (Microsoft Corp., Redmond, WA, USA) softwares.

Because we obtained four times of 3D volumes for each BM (pre-SRS and first to third post-SRS follow-ups), we extracted the image features of each volume initially and modelled the four features for treatment response prediction sequentially. For the feature extraction of each volume, we utilised a convolutional neural network (CNN) model with randomly initialised ResNet-34 as the backbone. As an independent comparison arm of the 3D CNN, we used a two-dimensional (2D) CNN for the image analysis, in which 2D patches were derived from three orthogonal slices of each 3D patch.

**Figure 2:**
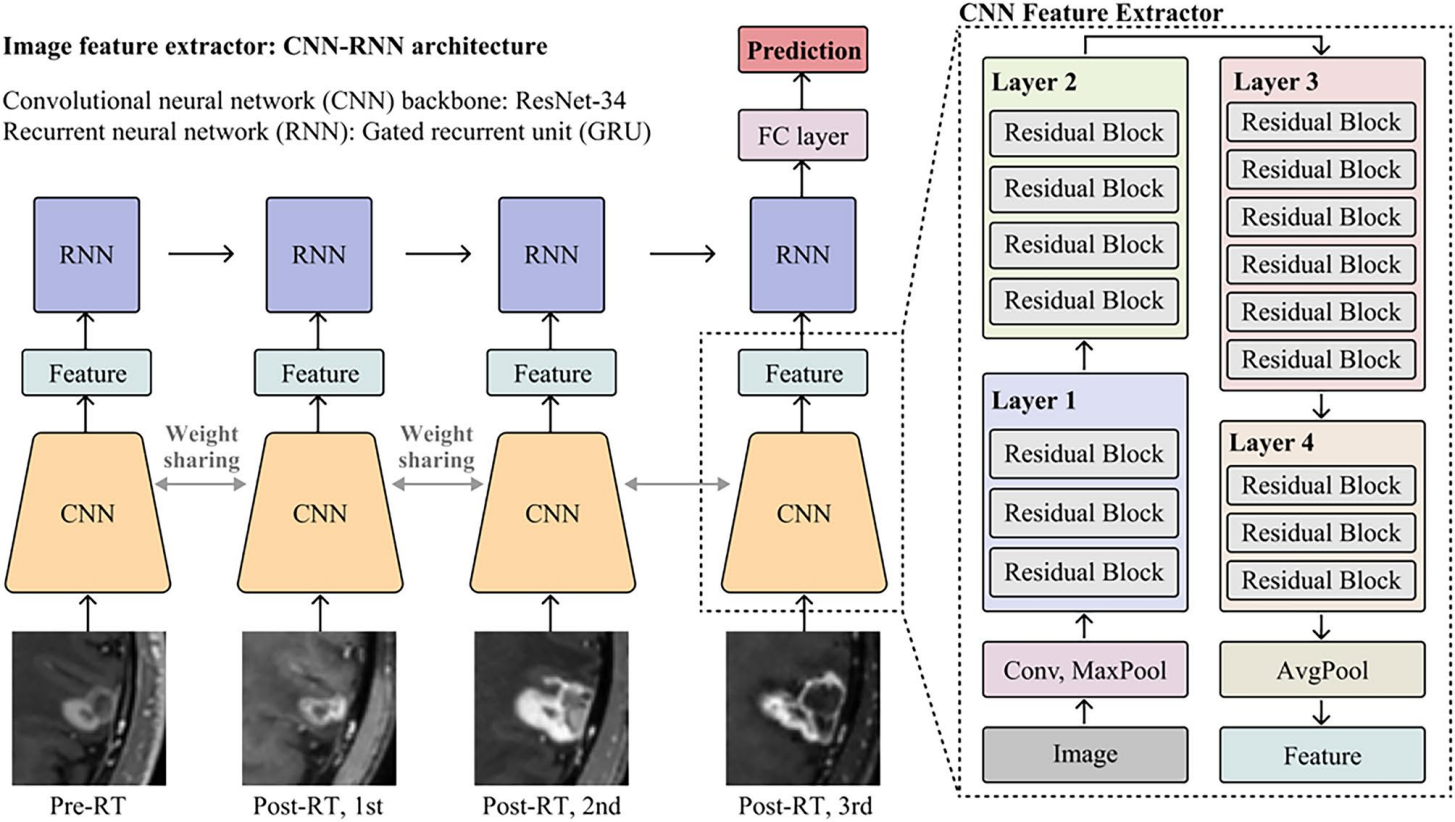
Model architecture of the proposed 2D convolutional neural network with a gated recurrent unit (Conv-GRU) model. *2D*, two-dimensional, *Conv-GRU* convolutional neural network with a gated recurrent unit.

We used two sequential modelling methods suitable for high-dimensional feature analysis for the preliminary model selection in the DL arm using the four image features from the CNN, each consisting of a 512-dimensional vector. First, we concatenated the four features and applied a fully connected layer, taking a 2048-dimensional vector as its input (simple CNN). Second, we used a gated recurrent unit (Conv-GRU) (Fig. 2)^23^, a deep neural network specialised in sequential modelling of high-dimensional deep features. The two models were trained in an end-to-end manner. Each simple CNN and Conv-GRU model for 10 distinct dataset splits (training:test = 8:2) was trained for the statistical analysis. We selected the model with the best accuracy after applying statistical analysis between the simple CNN and Conv-GRU. In addition, we conducted an ablation study by replacing the CNN and GRU components with other backbones in the 2D Conv-GRU model.

We applied data augmentation that comprised random rotation from -30° to + 30°, random scaling from 0.85 × to 1.15 × , random horizontal flip with 0.5 probability, and random translation from − 10 px to + 10 px for each axis. We used the Adam optimiser and the focal loss function for the learning hyperparameters and set the epoch, batch size, and learning rate to 20, 8, and 1 × 10^−4^, respectively. For cases where the epoch ranged between 10 and 15, we set the learning rate at 1 × 10^−5^. For cases in which the epoch was > 15, the learning rate was set at 1 × 10^−6^ to accommodate the higher epoch number.

To enhance the interpretability of the DL models, we conducted a post hoc analysis using a class activation map (CAM). This analysis highlighted the specific subregions of the input images from which the feature vector was extracted predominantly. Specifically, we utilised the Eigen-CAM algorithm, which improves the clarity of the CNN predictions by visualising the principal components of the learned representations from the convolutional layers^24^. For instance, a distinct activation pattern in an enhancing BM nodule boundary between PD and non-PD cases could reveal insights into the model’s classification ability.

As we sequentially modelled the CNN-extracted features with GRU, the Dmax and radiomics features of four different time points were analysed by the XGBoost models^25^. Specifically, the radiomics features, which consisted of the first-order statistics, shape, grey level co-occurrence, run length, and size zone matrices, were extracted using the PyRadiomics library^26^. Four Dmax and radiomics features of each patient were concatenated sequentially to formulate the input of the XGBoost models: input vectors were constructed by sequentially concatenating features (e.g. feature1 of time 1 to feature1 of time 4, or Dmax of time 1 to Dmax of time 4). By using all the features from four distinct time points, these approaches allowed models to assess feature importance and select relevant features automatically. The code for the implemented models in this study can be found in: https://github.com/w-cho/mri_convgru.

### Evaluation of model performance

First, we assessed the performance of each model in predicting binary treatment responses (PD versus non-PD). The prediction accuracies were obtained by training all four time points of the serial MRI scans (pre-SRS and first to third post-SRS follow-ups).

To identify the optimal number of follow-up MRI studies for predicting the treatment response after SRS, we evaluated the changes in prediction accuracy according to the number of serial (pre-SRS, pre-SRS to first post-SRS, pre-SRS to second post-SRS, and pre-SRS to third post-SRS) MRI scans for the best-performing model. We modified our Conv-GRU model architecture by increasing the number of sequential inputs from one to four while maintaining consistent experimental settings for dataset splitting, data augmentation, optimisation, loss function, epochs, batch size, and learning rate. The optimal hyperparameter for the focal loss was investigated for each fold using a grid search.

### Statistical analyses

The area under the curve (AUC), specificity, and sensitivity were assessed for each model. We derived the optimal cut-off values for the receiver-operating characteristic analysis from Youden’s J statistic. Post hoc tests were performed using the Bonferroni correction for multiple comparisons. We calculated the *P*-values of the prediction accuracy comparison using a paired *t*-test performed on the results of the individual splits in each model.

## Results

### Patient characteristics

Table 1 summarises the characteristics of the enrolled patients, primary cancer types, follow-up intervals between MRI examinations, maximum axial diameter of the BM nodules, and treatment responses as the ground truth. In the Developmental dataset, the enrolled patients (103 men, 91 women) had a mean age of 64.8 years. The mean number of BM nodules was two per patient. The predominant primary cancer types were lung cancer (74.2%), breast cancer (28%), kidney cancer (3.6%), colon cancer (3.6%), and ovarian cancer (1.5%). Furthermore, the bladder, oesophagus, pancreas, peritoneum, and ureter were primary cancer origin sites in only one case each. The mean intervals from the pre-SRS MRI to the first post-SRS MRI, first post-SRS MRI to the second post-SRS MRI, and second post-SRS MRI to the third post-SRS MRI were 2.6, 2.8, and 2.9 months, respectively. The total mean interval from the pre-SRS MRI to the third post-SRS MRI was 8.3 months. Among the 369 enrolled target BM nodules, 88 (23.8%) were classified as PD and 281 (76.2%) were assessed as non-PD, which consisted of 140 (37.9%) complete responses, 103 (27.9%) partial responses, and 38 (10.3%) stable disease according to the modified RANO-BM criteria.

**Table 1:** Demographic characteristics of included patients.

Characteristics	Developmental dataset	Temporal test set
	Patients (n = 194)	Patients (n = 43)
Female: Male, number (%)	103 (53.1): 91 (46.9)	20 (46.5): 22 (51.2)
Mean age at diagnosis ± SD, years	64.8 ± 11.1 (range, 27–95)	64 ± 10 (range, 38–85)
Number of BM nodules	2 (range, 1–10)	1 (range, 1–5)
Primary cancer type		
Lung, number (%)	144 (74.2, NSCLC: SCLC = 132:12)	31 (72.1, NSCLC: SCLC = 28:3)
Breast, number (%)	28 (14.4)	2 (4.7)
Kidney, number (%)	7 (3.6)	4 (9.3)
Colon, number (%)	7 (3.6)	3 (7)
Ovary, number (%)	3 (1.5)	NA
Ureter, number (%)	1 (0.5)	1 (2.3)
Bladder, number (%)	1 (0.5)	Others 3 (7; GB, Liver, Melanoma)
Oesophagus, number (%)	1 (0.5)	NA
Pancreas, number (%)	1 (0.5)	NA
Peritoneum, number (%)	1 (0.5)	NA
Follow up Intervals of MRIs, Mean F/U ± SD, months		
Pre-SRS–1st post-SRS	2.6 ± 0.66 (95% CI, 2.5–2.6)	2.9 ± 0.6 (95% CI, 2.8–3)
1st post-SRS–2nd post-SRS,	2.8 ± 1.3 (95% CI, 2.7–2.9)	2.9 ± 1.1 (95% CI, 2.7–3.1)
2nd post-SRS–3rd post-SRS	2.9 ± 1.2 (95% CI, 2.8–3)	3.1 ± 2 (95% CI, 2.8–3.4)
Pre-SRS–3rd post-SRS	8.3 ± 2.2 (95% CI, 8.1–8.5)	8.9 ± 2.7 (95% CI, 8.5–9.3)
1.5 T: 3 T number of MRIs		
On Pre-SRS	194: 0	43: 0
On 1st post-SRS	76: 118	19: 24
On 2nd post-SRS	75: 119	15: 28
On 3rd post-SRS	79: 115	17: 26
Nodular diameter, Mean ± SD, mm		
On Pre-SRS MRIs	14.1 ± 9 (range, 5–58)	12 ± 7 (range, 5–31)
On 1st post-SRS mris	9.3 ± 8 (range, 0–47)	7 ± 6 (range, 0–29)
On 2nd post-SRS mris	8.6 ± 8.6 (range, 0–52)	7 ± 7 (range, 0–24)
On 3rd post-SRS mris	8.5 ± 9.2 (range, 0–59)	7 ± 7 (range, 0–27)
Ground truth of treatment response	Nodules (n = 369)	Nodules (n = 62)
PD, number (%)	88 (23.8)	15 (24.2)
Non-PD, number (%)	281 (76.2)	47 (75.8)
Complete response	140 (37.9)	17 (27.4)
Partial response	103 (27.9)	22 (35.5)
Stable disease	38 (10.3)	8 (12.9)

*CI* confidence interval, *MRI* magnetic resonance imaging, *NSCLC* non-small cell lung cancer, *PD* progressive disease, *SCLC* small cell lung cancer, *SD* standard deviation, *SRS* stereotactic radiosurgery.

In the Temporal test set, the enrolled patients (22 men, 20 women) had a mean age of 64 years. The predominant primary cancer type was lung cancer (72.1%). The mean intervals from the pre-SRS MRI to the first post-SRS MRI, first post-SRS MRI to the second post-SRS MRI, and second post-SRS MRI to the third post-SRS MRI were 2.9, 2.9, and 3.1 months, respectively. The total mean interval from the pre-SRS MRI to the third post-SRS MRI was 8.9 months. Among the 62 enrolled target BM nodules, 15 (24.2%) were classified as PD and 47 (75.8%) were assessed as non-PD according to the modified RANO-BM criteria.

In both datasets, all patients underwent pre-SRS MRI using a 1.5-T MR scanner. The subsequent three MRI scans were chosen randomly from either a 1.5-T or 3-T MR scanner. The proportion of 1.5-T scans was consistent across both datasets, with a ratio of 0.55 (424/776 in the Developmental dataset and 94/172 in the Temporal test set).

### Performance comparison between the models

**Figure 3:**
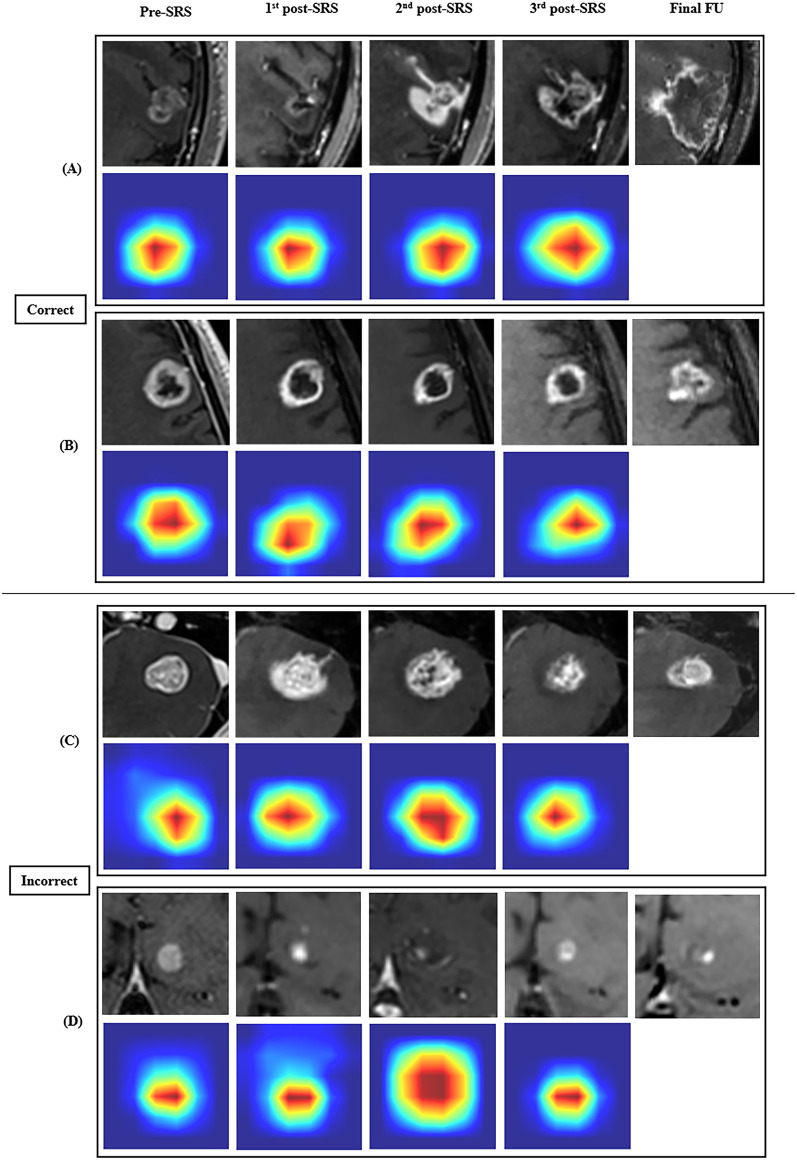
Representative cases that were correctly predicted (**A**,**B**) and incorrectly predicted (**C**,**D**) by the 2D Conv-GRU model. Each image consists of four sequential MRI scans, their corresponding Eigen-CAM images, and the most recent follow-up MRI scans used for establishing ground truth. (**A**) A PD case histopathologically confirmed via surgery 18 months post-SRS exhibited no significant change in the size between the second and third post-SRS follow-up MRIs. However, the corresponding Eigen-CAM image revealed stronger activation. The 2D Conv-GRU model correctly predicted PD. (**B**) A non-PD case showed stable disease on 12 months post-SRS. While the size remained stable on the third post-SRS follow-up MRI, the corresponding Eigen-CAM image displayed weaker activation than the second post-SRS follow-up MRI. The 2D Conv-GRU model accurately predicted non-PD. (**C**) A PD case, confirmed through surgery 17 months post-SRS, showed a decrease in size by the third post-SRS follow-up MRI. The corresponding Eigen-CAM image exhibited weaker activation, leading the 2D Conv-GRU model to mispredict non-PD. (**D**) A non-PD case remained stable up to 80 months post-SRS. The Eigen-CAM image for the second post-SRS follow-up MRI lost focus on the tumour, resulting in an incorrect PD prediction by the 2D Conv-GRU model. *2D* two-dimensional, *Conv-GRU* convolutional neural network with a gated recurrent unit, *MRI* magnetic resonance imaging, *CAM* class activation map, *PD* progressive disease, *SRS* stereotactic radiosurgery.

At the preliminary model selection level in the DL arm, the AUC of the Conv-GRU was superior to that of the simple CNN in 2D (0.8782 versus 0.8344; P < 0.001) and 3D (0.8311 versus 0.7918; P = 0.007) (Supplementary Tables 1 and 2). The results of ablation study for substituting CNN and GRU components with alternative architectures in the 2D Conv-GRU model are presented in Supplementary Table 3. For the Developmental dataset, the mean AUCs from the 10 distinct dataset splits were 0.8782, 0.8311, 0.8228, and 0.7483 for 2D Conv-GRU, 3D Conv-GRU, Dmax, and radiomics, respectively (Table 2). For the Temporal test set, the mean AUCs were 0.8341, 0.7836, 0.7516, and 0.7779 for 2D Conv-GRU, 3D Conv-GRU, Dmax, and radiomics, respectively (Supplementary Table 4). For the Developmental dataset, the mean AUC of the 2D Conv-GRU model was significantly higher than that of the 3D Conv-GRU, Dmax, and radiomics model (P = 0.0028, P < 0.0001, and P < 0.0001, respectively). The mean AUC of the 3D Conv-GRU model was significantly higher than that of the radiomics model (P = 0.0003). Finally, the mean AUC of the radiomics model was inferior to that of the Dmax model (P = 0.0015). For the Temporal test set, the mean AUC of the 2D Conv-GRU model was significantly higher than that of the 3D Conv-GRU, Dmax, and radiomics model (P = 0.0005, P < 0.0001, and P = 0.0002, respectively), similar to the finding of the Developmental dataset. The mean AUC of the radiomics model was also inferior to that of the Dmax model (P = 0.0086) (Table 3). In the representative case, the DL model accurately predicted the PD and non-PD cases, despite the temporal changes in solidity and diameter (Fig. 3). In cases where predictions were accurate, the model consistently concentrated on the enhancing BM nodule across all four MRI scans. Conversely, in cases of incorrect predictions, the model often shifted its attention away from the BM nodule. Additionally, viable tumour regions tended to show stronger activation, while areas of post-treatment change showed weaker activation.

**Table 2:** Predictive accuracy of models for assessing treatment response after stereotactic radiosurgery of brain metastasis.

Model	2D Conv-GRU			3D Conv-GRU			Dmax			Radiomics		
Split	AUC	Spec	Sens	AUC	Spec		AUC	Spec	Sens	AUC	Spec	Sens
1	0.8597	0.8256	0.8125	0.8241	0.8256	0.75	0.7972	0.5233	0.9375	0.7078	0.6047	0.8125
2	0.8845	0.9286	0.7059	0.8445	0.8214	0.7647	0.8466	0.625	0.9412	0.7805	0.6529	0.8235
3	0.8781	0.7636	0.8824	0.7968	0.6545	0.8235	0.7995	0.7818	0.7059	0.7743	0.4727	0.9412
4	0.8496	0.6809	0.8667	0.8	0.7872	0.7333	0.7915	0.9362	0.4667	0.7043	0.7021	0.6667
5	0.8355	0.9266	0.5714	0.801	0.625	0.8571	0.8099	0.75	0.7857	0.6511	0.6786	0.5714
6	0.8356	0.6275	0.8462	0.7994	0.4902	0.9231	0.7421	0.8431	0.5385	0.6757	0.8824	0.4615
7	0.9535	0.8837	0.9286	0.9269	0.814	0.9286	0.9286	0.8605	0.8571	0.799	0.6744	0.8571
8	0.8929	0.8182	0.8667	0.8202	0.7121	0.8	0.8485	0.7424	0.8	0.7793	0.7727	0.7333
9	0.8786	0.8571	0.75	0.7636	0.6286	0.85	0.8179	0.7286	0.75	0.7321	0.7286	0.75
10	0.9135	0.7714	0.9474	0.9346	0.7286	0.9474	0.8462	0.7286	0.7895	0.8793	0.7857	0.8421
**Mean**	0.8782	0.8083	0.8178	0.8311	0.7087	0.8378	0.8228	0.752	0.7572	0.7483	0.6955	0.7459
**SD**	0.0365	0.0996	0.1139	0.0566	0.1096	0.0772	0.0493	0.1176	0.1545	0.0675	0.1109	0.1445

*AUC* area under the receiver-operating characteristic curves, *Conv-GRU* convolutional neural network with a gated recurrent unit, *Dmax* prediction model based on maximum axial diameter, *SD* standard deviation, *Sens* sensitivity, *Spec* specificity, *2D* two-dimensional, *3D* three-dimensional.

**Table 3:** P-values of comparison between predictive accuracies of models for assessing treatment response after stereotactic radiosurgery of brain metastasis.

	Developmental dataset				Temporal test set			
	2D Conv-GRU	3D Conv-GRU	Dmax	Radiomics	2D Conv-GRU	3D Conv-GRU	Dmax	Radiomics
2D ConvGRU	–	0.0028*	< 0.0001*	< 0.0001*	–	0.0005*	< 0.0001*	0.0002*
3D ConvGRU	–	–	0.5364	0.0003 *	–	–	0.272	0.5423
Dmax	–	–	–	0.0015 *	–	–	–	0.0086
Radiomics	–	–	–	–	–	–	–	–

*P-values less than 0.0125 indicated a statistically significant difference after Bonferroni correction for 4-axis multiple comparison.

### Model performance comparison among the follow-up periods

**Figure 4:**
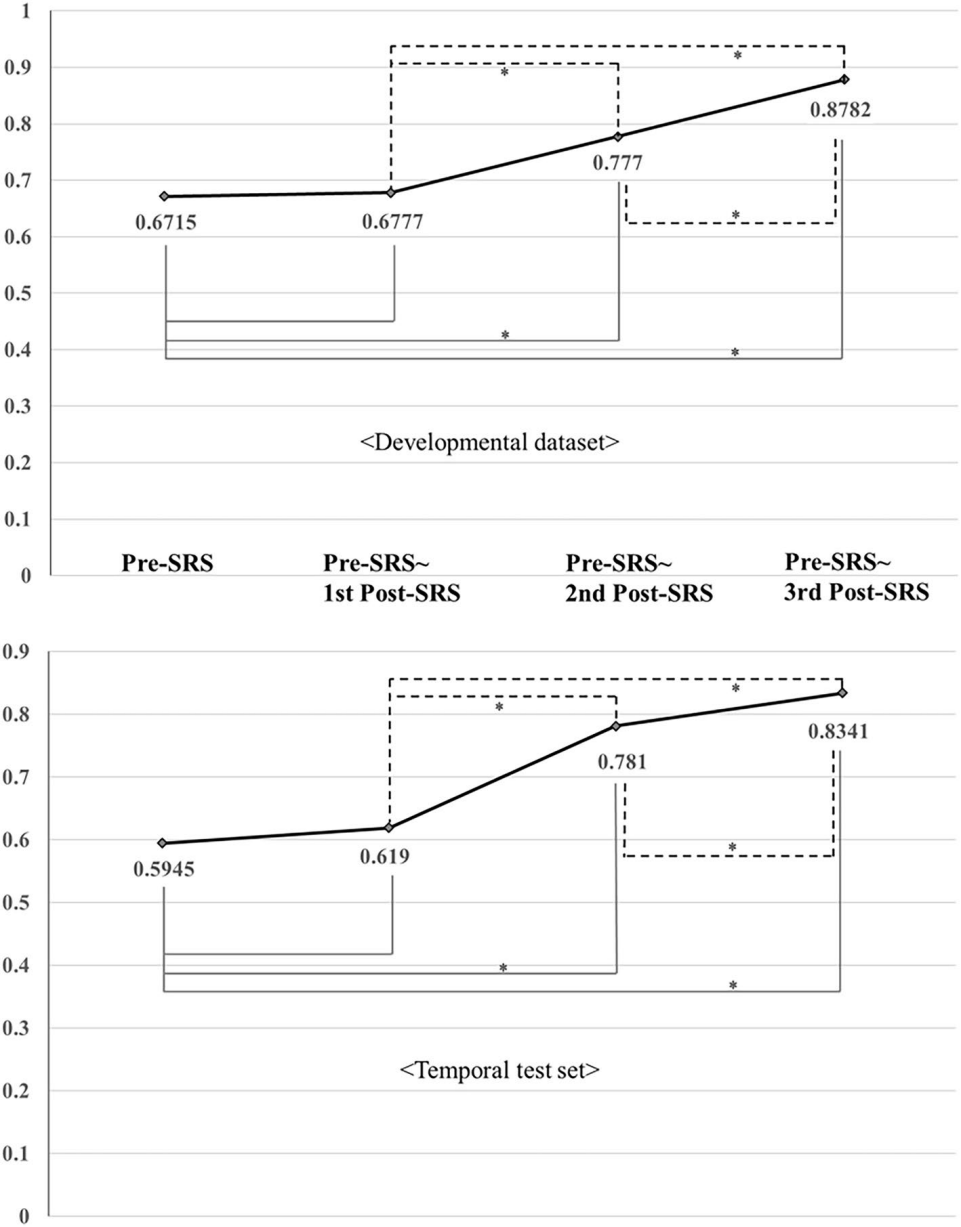
The area under the receiver-operating characteristic curves of the 2D Conv-GRU model with varying number of follow-up MRI scans. *Indicates a statistically significant difference. *2D* two-dimensional, *Conv-GRU* convolutional neural network with a gated recurrent unit, *MRI* magnetic resonance imaging.

For the Developmental dataset, the AUC pattern of the 2D Conv-GRU model displayed a gradual increment corresponding to the follow-up periods (AUC of 0.6715, 0.6777, 0.777, and 0.878; only pre-SRS MRI, plus 1, 2, and 3 post-SRS MRI(s), respectively). The AUC from the pre-SRS to the third post-SRS follow-up was significantly higher than that of the remaining periods (P < 0.0001). Additionally, the AUC from the pre-SRS to the second post-SRS follow-up was significantly higher than that from the pre-SRS only or from the pre-SRS to the first post-SRS follow-up (P < 0.0001). For the Temporal test set, the AUC of the 2D Conv-GRU model also improved incrementally with the addition of follow-up MRI scans (AUC of 0.5945, 0.6190, 0.7810, and 0.8341; only pre-SRS MRI, plus 1, 2, and 3 post-SRS MRI(s), respectively). Likewise, the utilisation of all four MRI scans resulted in a significantly higher AUC compared to analyses with fewer scans (P < 0.0001) (Fig. 4, Table 4).

**Table 4:** P-values of comparison with varying number of follow-up MRIs using 2D ConvGRU model for assessing treatment response after stereotactic radiosurgery of brain metastasis.

		Developmental dataset			Temporal test set		
		Pre-SRS ~ 1st Post-SRS	Pre-SRS ~ 2nd Post-SRS	Pre-SRS ~ 3rd Post-SRS	Pre-SRS ~ 1st Post-SRS	Pre-SRS ~ 2nd Post-SRS	Pre-SRS ~ 3rd Post-SRS
AUC	Pre-SRS	0.4971	< 0.0001*	< 0.0001*	0.1103	< 0.0001*	< 0.0001*
	Pre-SRS ~ 1st Post-SRS		< 0.0001*	< 0.0001*		< 0.0001*	< 0.0001*
	Pre-SRS ~ 2nd Post-SRS			< 0.0001*			0.0006*
Specificity	Pre-SRS	0.0273	0.4632	0.0013*	0.012	0.0008*	< 0.0001*
	Pre-SRS ~ 1st Post-SRS		0.2924	0.1149		0.9836	0.2976
	Pre-SRS ~ 2nd Post-SRS			0.0470			0.0743
Sensitivity	Pre-SRS	0.0451	0.3631	0.085	0.0033*	0.82	0.99
	Pre-SRS ~ 1st Post-SRS		0.0091*	< 0.0001*		0.01*	0.0134
	Pre-SRS ~ 2nd Post-SRS			0.5772			0.7844

* P-value less than 0.0125 indicated a statistically significant difference after Bonferroni correction for 4-axis multiple comparison.

## Discussion

This study used longitudinal MRI data to demonstrate the prediction performance for the treatment response after SRS of BM of the DL (2D versus 3D), radiomics, and Dmax models. The 2D Conv-GRU model displayed superior performance relative to that of the 3D Conv-GRU, radiomics, and Dmax models. Moreover, upon evaluating the 2D Conv-GRU model with varying follow-up periods, the prediction accuracy tended to increase with the number of follow-up MRIs.

Clinicians should consider the possibility of tumour progression and radiation necrosis upon observing an initial increase in tumour size or new contrast-enhancing lesions in the treated area after SRS. Despite their vastly different long-term outcomes, it can be challenging to distinguish between the two conditions in the early post-SRS period using conventional MRI^6^. This aspect is primarily attributed to early tumour size changes after SRS that do not always correlate with the long-term response. Several factors, including genetics, age, performance status, radiation dose or regimen, tumour number or size, and histopathology, may contribute to confusion while assessing the treatment response^5,27,28^, thereby delaying confirmative assessment and timely treatment^8^. Whereas advanced MRI techniques, such as diffusion-weighted imaging, perfusion-weighted imaging, and spectroscopy, as well as positron emission tomography, have been evaluated to supplement conventional MRI, they have not yet demonstrated promising results^29–32^. As such, the RANO-BM working group recommends a multidisciplinary team decision-making process to assess the treatment response instead of relying on a single modality^6^.

A recent systematic review and meta-analysis suggested that the performance of AI-assisted MRI in classifying tumour progression and radiation necrosis after radiotherapy of BM is inadequate for clinical use^18^. The authors identified several issues, such as the need for extensive DL research, consecutive data recruitment that reflects real-world clinical settings, larger sample sizes for robustness, and research using MRI data from multiple time points. Only a limited number of studies have been published on this topic, and the reported performance remains insufficient. Specifically, one study demonstrated AUCs of 0.72 for DL alone and 0.80 for combined DL and radiomics models^17^, highlighting the need for further improvement. Additionally, BM is the most common brain malignancy in adults, and it is relatively easy to obtain a large sample size; therefore, DL research may be a more suitable methodology than radiomics. Multiparametric evaluation is another research trend, which has presented predictive AUCs from 0.71 to 0.86^15,16^. These researchers co-registered multiple MRI sequences into a single template to combine the information, thus enhancing predictive accuracy. However, they typically use single time point MRI data, which are not representative of daily clinical practice.

In addition, few studies have investigated the use of longitudinal MRI analysis to assess the treatment response of BM^33,34^. This phenomenon is primarily attributed to the difficulty in obtaining longitudinal datasets for BM because the size of the dataset is multiplied by the length of the follow-up period. Nevertheless, the treatment response is assessed based on the serial follow-up MRI scans; accordingly, the model should use data from multiple time points for accurate prediction, rather than relying on that from a single time point. Cho et al.^33^ developed and validated a DL model to assess automated treatment response using the RANO-BM criteria; however, the model was designed to provide the current treatment response rather than to predict the *future* treatment response. Lee et al.^34^ conducted a tumour habitat analysis using longitudinal MRI data to predict tumour recurrence after SRS. Using a k-means clustering algorithm, they classified each tumour tissue on physiologic MR images (composed of apparent diffusion coefficient and cerebral blood volume images) into nonviable tissue, hypovascular cellular, and hypervascular cellular habitats. Based on the differences between the first and second follow-up MRI scans, an increase in the hypovascular cellular habitat was the most strongly associated with tumour recurrence.

In this novel study, we applied DL models to analyse longitudinal MRI data from more than two time points to predict the BM treatment response. The 2D Conv-GRU model outperformed the radiomics and Dmax models using four-point sequential MRI data from both the Developmental dataset and Temporal test set. This result suggests that CNN encoders can extract more comprehensive information from MRI than handcrafted feature extraction methods, such as Dmax and radiomics, can. In other words, DL models can automatically extract the most relevant features from MRI scans for treatment response prediction. Moreover, the GRU-based decoder in our DL model, which sequentially acquires multiple inputs, effectively handles sequential data, leading to superior results in the longitudinal MRI analysis.

The accuracy of the DL model increased gradually as the number of follow-up studies increased, highlighting the importance of longitudinal assessments. However, the trend of the performance increment did not reach a plateau even when using all four time points (Fig. 4).Therefore, extending the observation period beyond four time points may further improve the prediction accuracy, which warrants further investigation.

In this study, we used a modified version of the RANO-BM criteria, which permitted the consideration of BM nodules as small as 5 mm as measurable lesions, which was suggested by the RANO-BM working group^6^. Advances in MRI hardware have facilitated using thin section images (≤ 1.5 mm) for BM evaluation. This modification increases the number of measurable lesions, potentially resulting in greater reliability of the treatment response assessment. Previous computer-aided detection studies using MRI data reported a mean maximum diameter of < 1 cm (5–9 mm) of the BM nodules^35–39^. Hence, adopting a size threshold of 5 mm is reasonable.

This study had some limitations. First, while we conducted an external validation with temporally separated data, we did not utilise data from other institutions. In addition, the relatively small sample size for model training may not sufficiently capture the temporal dynamics of the data. Consequently, we plan to conduct a follow-up multicentre study to evaluate the generalisability of our model. Second, the ground truth was based principally on clinical and radiological information, with only a few cases confirmed by histopathological evaluation. Despite being a common limitation in similar retrospective studies, it may have affected the accuracy of our results. The retrospective design of our study also may have introduced selection bias. Third, this study included MRI scans obtained from both 1.5-T and 3-T scanners, which introduces potential biases due to the inherent differences in image quality and characteristics. However, we noted an even distribution of patient scans across each dataset, which might have neutralised and mitigated the potential biases by a randomisation effect. Fourth, the effect of the follow-up interval between the MRI scans on the results cannot be entirely excluded, despite the small standard deviations of the intervals. Finally, the requirement for pre-processing and segmentation poses a significant challenge to its clinical applicability. Streamlining this process through integration with our picture archiving and communication system could offer substantial benefits.

In conclusion, using longitudinal MRI data, the 2D Conv-GRU model outperformed the 3D Conv-GRU, radiomics, and Dmax models in predicting the treatment response after SRS of BM. Our results suggest that using three post-SRS MRI examinations can achieve the best performance.

### Supplementary Information

Supplementary Information.

## Data Availability

The code for the implemented models in this study can be found in: https://github.com/w-cho/mri_convgru. The datasets presented in this article are not readily available because they are subject to the permission of the Institutional Review Board of the participating institution. Requests to access the datasets should be directed to leonard.sunwoo@gmail.com.
