# Supplementary material for: Prediction of treatment response after stereotactic radiosurgery of brain metastasis using deep learning and radiomics on longitudinal MRI data
Source: Sci Rep. 2024 May 15;14:11085. doi: 10.1038/s41598-024-60781-5 (PMC11096355; doi:10.1038/s41598-024-60781-5)
Supplement: Supplementary file 1 — Supplementary Information. [file 41598_2024_60781_MOESM1_ESM.pdf]

Prediction of treatment response after stereotactic radiosurgery of brain metastasis using deep learning and radiomics on longitudinal MRI data

Se Jin Cho[1†], Wonwoo Cho[2,3†], Dongmin Choi[2,3], Gyuhyeon Sim[2,3], So Yeong Jeong[1], Sung Hyun Baik[1], Yun Jung Bae[1], Byung Se Choi[1], Jae Hyoung Kim[1], Sooyoung Yoo[4], Jung Ho Han[5], Chae-Yong Kim[5], Jaegul Choo[2,3*] & Leonard Sunwoo[1,6*]

[1]Department of Radiology, Seoul National University Bundang Hospital, Seoul National University College of Medicine, 82, Gumi-ro 173beon-gil, Bundang-gu, Seongnam, Gyeonggi 13620, Republic of Korea

[2]Kim Jaechul Graduate School of Artificial Intelligence, KAIST 291 Daehak-ro, Yuseong-gu, Daejeon 34141, Republic of Korea

[3]Letsur Inc., 180 Yeoksam-ro, Gangnam-gu, Seoul 06248, Republic of Korea

[4]Office of eHealth Research and Business, Seoul National University Bundang Hospital, 82, Gumi-ro 173beon-gil, Bundang-gu, Seongnam, Gyeonggi 13620, Republic of Korea

[5]Department of Neurosurgery, Seoul National University Bundang Hospital, Seoul National University College of Medicine, 82, Gumi-ro 173beon-gil, Bundang-gu, Seongnam, Gyeonggi 13620, Republic of Korea

[6]Center for Artificial Intelligence in Healthcare, Seoul National University Bundang Hospital, 82, Gumi-ro 173beon-gil, Bundang-gu, Seongnam, Gyeonggi 13620, Republic of Korea

[†]Se Jin Cho and Wonwoo Cho contributed equally to this work.

[*]Jaegul Choo and Leonard Sunwoo contributed equally to this work.

***Co-corresponding authors:**

Jaegul Choo, PhD

Kim Jaechul Graduate School of Artificial Intelligence, KAIST 291 Daehak-ro, Yuseong-gu, Daejeon 34141, Republic of Korea and Letsur Inc., 180 Yeoksam-ro, Gangnam-gu, Seoul 06248, Republic of Korea

E-mail: jchoo@kaist.ac.kr; Tel: +82-42-350-1813; Fax: +82-42-350-1813

Leonard Sunwoo, MD, PhD

Department of Radiology, Seoul National University Bundang Hospital, 82, Gumi-ro 173beon-gil, Bundang-gu, Seongnam, Gyeonggi 13620, Republic of Korea

E-mail: leonard.sunwoo@gmail.com; Tel: +82-31-787-7631; Fax: +82-31-787-4011

Supplementary Table 1. Preliminary evaluation of accuracy of both simple CNN and Conv-GRU for model selection using 2D patch.

	**Simple 2D CNN**			**2D Conv-GRU**			
**Split**	**AUC**	**Spec**	**Sens**	**AUC**	**Spec**	**Sens**	*P-value*
1	0.7965	0.814	0.6250	0.8597	0.8256	0.8125	
2	0.8487	0.8214	0.8235	0.8845	0.9286	0.7059	
3	0.8813	0.8727	0.7647	0.8781	0.7636	0.8824	
4	0.8213	0.8298	0.6667	0.8496	0.6809	0.8667	
5	0.7943	0.7143	0.7857	0.8355	0.9266	0.5714	
6	0.7722	0.6471	0.7692	0.8356	0.6275	0.8462	
7	0.9070	0.6977	0.9286	0.9535	0.8837	0.9286	
8	0.8362	0.8333	0.6667	0.8929	0.8182	0.8667	
9	0.8250	0.8714	0.7500	0.8786	0.8571	0.7500	
10	0.8617	0.7571	0.8947	0.9135	0.7714	0.9474	
**Mean**	**0.83442**	0.7859	0.7675	**0.8782**	0.80832	0.8178	*< 0.0001*
**SD**	0.04163	0.0775	0.0981	0.0365	0.09956	0.1139	

Abbreviations: AUC, area under the receiver-operating characteristic curve; CNN, convolutional neural network; Conv-GRU, convolutional neural network with a gated recurrent unit; SD, standard deviation; Sen, sensitivity; Spec, specificity; 2D, two-dimensional

Supplementary Table 2. Preliminary evaluation of accuracy of both simple CNN and Conv-GRU for model selection using 3D patch.

	**Simple 3D CNN**			**3D Conv-GRU**			
**Split**	**AUC**	**Spec**	**Sens**	**AUC**	**Spec**	**Sens**	*P-value*
1	0.7594	0.6395	0.7500	0.8241	0.8256	0.7500	
2	0.7910	1.000	0.4706	0.8445	0.8214	0.7647	
3	0.7754	0.8364	0.6471	0.7968	0.6545	0.8235	
4	0.7844	0.5532	0.8667	0.8000	0.7872	0.7333	
5	0.7870	0.9107	0.5000	0.8010	0.625	0.8571	
6	0.8235	0.7255	0.8462	0.7994	0.4902	0.9231	
7	0.8937	0.7209	0.9286	0.9269	0.814	0.9286	
8	0.7222	0.5758	0.8667	0.8202	0.7121	0.8000	
9	0.725	0.8286	0.4500	0.7636	0.6286	0.8500	
10	0.8564	0.7571	0.8947	0.9346	0.7286	0.9474	
**Mean**	**0.7918**	0.7548	0.7221	**0.8311**	0.70872	0.8378	*0.007*
**SD**	0.0540	0.1431	0.1893	0.0566	0.1096	0.0772	

Abbreviations: AUC, area under the receiver-operating characteristic curve; CNN, convolutional neural network; Conv-GRU, convolutional neural network with a gated recurrent unit; SD, standard deviation; Sen, sensitivity; Spec, specificity; 3D, three-dimensional

Supplementary Table 3. Results of ablation study substituting CNN and GRU components with alternative architectures in the 2D Conv-GRU model.

	**Developmental dataset**			**Temporal test set**		
**Model**	AUC	Spec	Sens	AUC	Spec	Sens
**ResNet34 + GRU (ours)**	0.8782 ± 0.0364	0.8083 ± 0.0996	0.8178 ± 0.1139	0.8341 ± 0.0197	0.7717 ± 0.0935	0.7533 ± 0.0835
**ResNet18 + GRU**	0.8492 ± 0.0402	0.7862 ± 0.0246	0.7807 ± 0.0379	0.8168 ± 0.0338	0.7570 ± 0.0436	0.7371 ± 0.0480
**ResNet50 + GRU**	0.8035 ± 0.0354	0.7479 ± 0.0431	0.7328 ± 0.0337	0.7854 ± 0.0364	0.7485 ± 0.0320	0.7127 ± 0.0297
**ResNet34 + RNN**	0.8256 ± 0.0362	0.7658 ± 0.0491	0.7944 ± 0.0495	0.7907 ± 0.0338	0.7293 ± 0.0452	0.7473 ± 0.0403
**ResNet34+ LSTM**	0.8661 ± 0.0272	0.7823 ± 0.0398	0.8022 ± 0.0429	0.8283 ± 0.0135	0.7596 ± 0.0151	0.7660 ± 0.0203

Values are mean ± standard deviation. Abbreviations: AUC, area under the receiver-operating characteristic curve; CNN, convolutional neural network; Conv-GRU, convolutional neural network with a gated recurrent unit; GRU; gated recurrent unit; LSTM, long short-term memory; RNN, recurrent neural network; Sens, sensitivity; Spec, specificity; 2D, two-dimensional.

Supplementary Table 4. Predictive accuracy of models for assessing treatment response after stereotactic radiosurgery of brain metastasis in the Developmental dataset.

**Model**	**2D Conv-GRU**			**3D Conv-GRU**			**Dmax**			**Radiomics**		
**Split**	**AUC**	**Spec**	**Sens**	**AUC**	**Spec**	**Sens**	**AUC**	**Spec**	**Sens**	**AUC**	**Spec**	**Sens**
1	0.8261	0.7609	0.7333	0.6464	0.9565	0.3333	0.7362	0.7447	0.7333	0.7546	0.7234	0.8000
2	0.8058	0.7174	0.8000	0.6754	0.6304	0.6667	0.734	0.8085	0.6667	0.7426	0.6383	0.8667
3	0.8087	0.8043	0.6667	0.8464	0.7609	0.7333	0.7745	0.766	0.7333	0.8071	0.6809	0.8667
4	0.8217	0.5870	0.8667	0.8667	0.6957	0.8667	0.766	0.8298	0.7333	0.7766	0.7234	0.8000
5	0.8333	0.8043	0.7333	0.8304	0.8043	0.7333	0.7092	0.766	0.6667	0.7716	0.766	0.7333
6	0.8420	0.8478	0.7333	0.8058	0.6957	0.8000	0.7333	0.7872	0.6667	0.7787	0.8298	0.7333
7	0.8406	0.8043	0.7333	0.8435	0.8696	0.6667	0.7291	0.7021	0.7333	0.7411	0.6809	0.7333
8	0.8667	0.6957	0.8667	0.7580	0.6739	0.7333	0.7759	0.8511	0.6000	0.8248	0.8511	0.8000
9	0.8594	0.9348	0.6000	0.7130	0.8696	0.5333	0.7865	0.8511	0.6000	0.766	0.7234	0.8000
10	0.8362	0.7609	0.8000	0.8507	0.7174	0.8000	0.7716	0.766	0.6667	0.8156	0.7021	0.8667
**Mean**	**0.8341**	0.7717	0.7533	**0.7836**	0.7674	0.6867	**0.7516**	0.7873	0.68	**0.7779**	0.73193	0.8000
**SD**	0.0197	0.0935	0.0835	0.0801	0.1045	0.1541	0.0261	0.0481	0.0526	0.0294	0.0667	0.05446

Abbreviations: AUC, area under the receiver-operating characteristic curves; Conv-GRU, convolutional neural network with a gated recurrent

unit; Dmax, prediction model based on maximum axial diameter; SD, standard deviation; Sens, sensitivity; Spec, specificity; 2D, two-dimensional; 3D, three-dimensional.

Glossary of deep learning terms

Convolutional neural network (CNN): A type of deep learning model highly effective for processing data with a grid-like topology, such as images. CNNs use convolutional layers to automatically and adaptively learn spatial hierarchies of features from input images, making them powerful for tasks like image recognition.

Recurrent neural network (RNN): A class of neural networks designed to handle sequential data, such as time series or text. RNNs can capture temporal dependencies because they process data in sequence, allowing the output from one step to influence the input to the next step.

Gated recurrent unit (GRU): A variant of RNN that aims to solve the vanishing gradient problem of traditional RNNs. GRUs use gating mechanisms to control the flow of information, making them more effective at capturing long-term dependencies in sequence data.

Conv-GRU: A hybrid neural network that combines convolutional layers with GRU layers. This architecture leverages the spatial feature extraction capabilities of CNNs and the temporal processing capabilities of GRUs, making it suitable for tasks that involve spatial-temporal data, such as video analysis.

Class activation mapping (CAM): A technique that generates heatmaps of class-specific areas by using the spatial information preserved in the convolutional layers of CNNs. CAM helps in understanding which parts of an image are important for making a classification decision, enhancing the interpretability of CNN models.

eXtreme Gradient Boosting (XGBoost): A highly efficient and scalable implementation of gradient boosting, a machine learning technique that builds models in a stage-wise fashion. XGBoost is known for its performance and speed, often used for classification, regression, and ranking tasks.

Radiomics: A field that involves the extraction of large amounts of quantitative features from medical images using data-characterisation algorithms. These features, which can describe tumour intensity, shape, texture, etc., are used to develop predictive models that can assist in clinical decision-making.

Technical details of our model and preprocessing steps

MRI Pre-processing Methodology

The pre-processing of magnetic resonance imaging (MRI) data encompasses several critical steps to ensure the accuracy and reliability of subsequent analyses. Detailed below are the methodologies employed:

BM Segmentation Annotation: Initial annotation of BM segmentation is conducted by two experienced neuroradiologists to ensure high precision and consistency.

Image Resampling for Uniform Voxel Spacing: To standardise the spatial resolution across all MRI scans, images are resampled to achieve a uniform voxel spacing of $0.5\times0.5\times0.5$ mm^3.

Signal Intensity Normalisation: The signal intensity of the resampled images is normalised to a range of -1 to 1, excluding the background. This normalisation is based on manually selected signal intensity values within the grey matter. Such standardisation is essential for mitigating variations in signal intensity that may arise from different MRI scanners or settings.

Extraction of BM Sub-region 3D Patches: 3D patches (dimensions: $64\times96\times96$) of BM subregions are extracted from the MRI data using the BM segmentation. Each BM sub-region is strategically positioned at the centre of its respective patch.

2D Slice Selection for Analysis: For analyses requiring a 2-dimensional representation, three slices are extracted from each 3D patch. This selection includes a central slice and two additional slices, chosen at predetermined intervals to either side of the centre. This methodical selection is designed to provide a comprehensive cross-sectional analysis of the BM subregions.

Technical specifications

We analysed 2D and 3D images using corresponding CNN models, specifically adopting the ResNet-34 architecture as the backbone for its efficacy in feature extraction across image dimensions.

ResNet-34 features a streamlined architecture of 34 layers, including 16 key residual blocks spread across four groups (3, 4, 6, and 3 blocks in each), leveraging skip connections for gradient flow. Each block contains two 3x3 convolutional layers, batch normalisation, and ReLU activation, efficiently tackling the vanishing gradient problem. This setup allows for deep network training, starting with an initial convolutional layer and ending with global average pooling and a fully connected layer for classification.

The output of ResNet-34 is a 512-dimensional feature vector. For longitudinal analysis of nodules, we use concatenation of these vectors followed by a fully connected layer or a GRU-based method.

The fully connected layer maps a 2048-dimensional input (512*4) to a 2-dimensional output, simplifying the feature space for classification.

The GRU configuration employs a hidden dimension of 256, a single layer, and a dropout

probability of 0.1 to capture temporal dynamics in the data, offering a sophisticated approach to nodule analysis.

Training Details

Optimizer: We employed the Adam optimizer for its adaptability and efficiency, which is particularly beneficial for our data's characteristics.

Batch Size: A batch size of 16 was chosen to balance computational demands with gradient stability.

Learning Rate and Epochs: We started with an initial learning rate of 1e-4, adjusting it downward by a factor of 0.1 at the 10th and 15th epochs, out of 20 epochs. This decay strategy helps fine-tune the model.

Loss Function: The Focal Loss function, with an alpha parameter of 0.1, was selected to effectively handle class imbalance, a common issue in medical datasets.
